# The parasitoid complex of *D. suzukii* and other fruit feeding Drosophila species in Asia

**DOI:** 10.1038/s41598-018-29555-8

**Published:** 2018-08-07

**Authors:** Pierre Girod, Nicolas Borowiec, Matthew Buffington, Guohua Chen, Yuan Fang, Masahito T. Kimura, Francisco Javier Peris-Felipo, Nicolas Ris, Hao Wu, Chun Xiao, Jinping Zhang, Alexandre Aebi, Tim Haye, Marc Kenis

**Affiliations:** 1 CABI, Delemont, Switzerland; 2 Laboratory of Fundamental and Applied Research in Chemical Ecology (FARCE), Univ. Neuchâtel, Faculté des Sciences, Neuchatel, Switzerland; 3 INRA, Univ. Nice Côte d’Azur, CNRS, UMR 1355 “Institut Sophia Agrobiotech”, Sophia Antipolis, France; 4 Systematic Entomology Laboratory, USDA Agricultural Research Service, Washington, D.C., USA; 5 College of Plant Protection, Yunnan Agricultural University, Kunming, China; 6 Hokkaido University Museum, Hokkaido University, Sapporo, Japan; 7 Bleichestrasse 15, CH–4058 Basel, Switzerland; 8 MoA-CABI Joint Laboratory for Bio-Safety, Institute of Plant Protection Chinese Academy of Agricultural Sciences, Beijing, China; 9 Laboratory of Soil Biodiversity, Univ. Neuchâtel, Faculté des Sciences, Neuchatel, Switzerland

## Abstract

*Drosophila suzukii* is an invasive fly of East Asian origin that has become a serious fruit pest worldwide. Classical biological control through the introduction of parasitoids from Asia could help reduce populations of *D. suzukii* in invaded regions. Little is known about the native parasitoids of the fly in Asia. Therefore, surveys for larval parasitoids of *D. suzukii* were carried out in China and Japan between 2015 and 2017. Parasitoids of *D. suzukii* and other fruit-inhabiting drosophilids (*D. pulchrella* and *D. subpulchrella*) that are probably attacked by the same parasitoid complex were found in four Chinese provinces and four Japanese prefectures. Larval parasitoids were obtained at most sites where *D. suzukii* was found, with parasitism varying from 0.0 to 75.6%. At least eight parasitoid species were reared. The most abundant and frequent parasitoids were the Figitidae *Ganaspis cf. brasiliensis* and *Leptopilina japonica*, but another *Leptopilina* species and at least five Braconidae species belonging to the genera *Areotetes*, *Asobara* and *Tanycarpa* were obtained in low numbers. Due to its likely restricted host range, the most promising parasitoid for biological control is *Ganaspis cf. brasiliensis*. However, its exact specificity and taxonomic status require future research.

## Introduction

The spotted wing Drosophila, *Drosophila suzukii* Matsumura (Diptera, Drosophilidae), is native to Asia and has recently invaded several regions and continents including Europe, North and South America, Reunion Island and Central Asia^1,2^. The economic impact of this invasive fly is increasing proportionally to its geographic range. Unlike most other Drosophilidae, *D. suzukii* is able to lay eggs in fresh fruits, due to the possession of a serrated ovipositor. With this feature, *D. suzukii* has become a major pest of small and stone fruits in most invaded regions^3–5^. It also has a very wide host range comprising many cultivated fruits as well as fruits from ornamental and wild plants^6–8^, and a short development time, which allows the completion of several generations per year^1^. As a result, crops are constantly reinvaded from neighbouring habitats, which complicates management strategies. Current control methods include chemical treatments and good cultural practices (e.g. sanitation, bait traps and insect proof nets)^9^. In addition, in invaded regions, *D. suzukii* encounters very few competitors. It is attacked by generalist predators^10^ and, to a much lower extent, by generalist pupal parasitoids^11–14^. In contrast, larval parasitoids, which are often considered as major mortality factors in Drosophilidae^15,16^, are so far absent from the natural enemy complex of *D. suzukii* in invaded regions^8,9,17^. Indeed, larval parasitoids of local Drosophilidae either do not show interest for *D. suzukii* or are not able to develop successfully in *D. suzukii* larvae, partly because of the strong host immune response of the fly larvae^18–22^.

Classical biological control, i.e. the introduction of larval parasitoids from the region of origin of the pest that are specialised in parasitizing *D. suzukii*, could help reduce populations at the landscape level and, consequently, decrease the need for management. However, little is known of the larval parasitoids of *D. suzukii* in Asia. The most comprehensive studies have recently been carried out in Japan but these often focused on specific parasitoid species or genera and rarely provided quantitative data on the role of parasitoids in the natural control of *D. suzukii*^23–27^. They concluded that the most promising biological control agent would be the figitid *Ganaspis brasiliensis* (Ihering) (Hymenoptera, Figitidae), a species that has been of unclear taxonomic status (first named *G. xanthopoda*^24^, and then *G. brasiliensis*^26,27^, following Buffington and Forshage^28^). This species is composed of different host races, some of which may warrant species status, and a specific host race appears to be specialised on *D. suzukii*^26^. In Asia, data on larval parasitism are restricted to a parasitoid survey in South Korea^29^ and a survey of the braconid genus *Asobara* in the Yunnan Province of China and South Korea^30^. Both studies used fruit collection and traps baited with uninfested fruits and suggest that field collection of suitable fresh fruits is a more reliable method to collect parasitoids. The most abundant larval parasitoids collected in South Korea were *Asobara japonica* Belokobylskij (Hymenoptera, Braconidae), *Ganaspis brasiliensis* and *Leptopilina japonica* Novković & Kimura (Hymenoptera, Figitidae)^29^.

In this publication, we report on surveys made from 2015 to 2017 in 12 Chinese Provinces and five Japanese prefectures to gather quantitative data on larval parasitism of *D. suzukii*. These surveys were made by sampling potentially suitable fresh fruits and, thus, also allowed us to collect two other *Drosophila* spp. living in the same habitat, *D. pulchrella* and *D. subpulchrella* Takamori and Watabe. The parasitoid complex of these two latter species is presently unknown.

## Results and Discussion

### Parasitism of *Drosophila* species

Details of all collections are provided in Table S1 (Supplementary information S1) and the results of the larval parasitoid surveys, with parasitism rates, are summarised in Table 1. For 2015 and 2016, only parasitism rates from samples that produced at least one larval parasitoid are shown because, in some cases, a high mortality occurred in the host pupal stage and the lack of parasitism may be due to sample deterioration in the period between fly and parasitoid emergence. For 2017, samples without parasitoid emergence were added because the rearing techniques had improved and we were more confident that all samples could be kept clean and healthy until parasitoid emergence. In any case, parasitism rates provided in Table 1 should be considered with caution. These rates are based on fly and parasitoid adult emergence and the rearing and transport conditions experienced during these surveys may have affected *D. suzukii* and its parasitoids very differently. On the one hand, most parasitoids emerge about two weeks after the *D. suzukii* adults. During this period, fruits became frequently covered by fungi, which surely prevented some parasitoids from emerging. On the other hand, *D. suzukii* pupae are extremely sensitive to high temperatures and low humidity. Unpublished observations by the authors showed that no fly emerges when pupae are exposed to temperature above 30 °C or humidity below 50% RH. It is possible that parasitoids in the host pupa are less sensitive than their host, however, nothing is known yet about the developmental requirements of the parasitoid species.

**Table 1:** Larval parasitoids and parasitism rates observed from fruit collections in China and Japan in 2015, 2016 and 2017.

Locality	Fruit	Month of collection	Other *Drosophila* sp. (%)^*^	Larval parasitoids and parasitism rates (%)						
				*Ganaspis* cf. *brasiliensis*	*Leptopilina japonica*	*Leptopilina* sp.	*Asobara* spp.^†^	*Tanycarpa* chors	*Areotetes striatiferus*	Total parasitism (n insects)
**China - Yunnan**										
Kunming - YAU	*Prunus cerasoides*	June 2015	5.1 (Dp)	42.0	10.9	—	—	—	—	52.9 (174)
Shiping	*Myrica rubra*	June 2015	3.5 (Dp)	1.7	0.7	—	—	—	—	2.4 (537)
Dali	*Prunus cerasoides*	May 2016	1.8 (Dp)	1.8	—	—	—	—	—	1.8 (224)
Kunming – West Mountain	*Prunus* (*Cerasus*) sp.	May 2016	6.7 (Dp)	1.4	—	—	—	—	—	1.4 (142)
Panzihua	*Prunus* (*Cerasus*) sp.	Apr 2016	0.0	2.1	—	—	—	—	—	2.1 (373)
Fumin	*Myrica rubra*	July 2016	74.5 (Dp)	0.4	11.8	35.9	6	—	—	54.1 (566)
Wenshan	*Myrica rubra*	June 2016	0.0	12.6	34.5	—	—	—	6.9	54.0 (87)
Kunming – West Mountain	*Myrica rubra*	July 2016	0.0	25.0	17.2	7.2	1.8	—	—	51.2 (615)
Dali	*Sambucus williamsii*	July 2016	55.3 (Dp)	1.2	—	—	1.0	—	—	2.3 (483)
Kunming - Snake Mountain	*Solanum nigrum*	July 2016	0.0	5.9	5.9	—	29.4	—	—	41.2 (17)
Wenshan	*Rubus* sp.	Sept 2016	73.5 (Dp)	2.0	7.0	—	—	—	—	9 (199)
Qujing	*Lonicera maacki*	Sept 2016	83.7 (Dp)	1.8	1.8	—	—	—	0.6	4.3 (326)
Kunming	*Lonicera maacki*	Sept 2016	65.0 (Dp)	8.7	4.3	—	—	—	—	13 (23)
Kunming – West mountain	*Prunus* (*Cerasus*) sp.	May 2017	14.0 (Dp)	—	—	—	—	—	—	0 (150)
Kunming - Snake mountain	*Rubus* sp.	May 2017	26.7 (Dp)	—	—	—	—	—	—	0 (56)
Kunming - YAU	*Rubus ellipticus* (?)	May 2017	90.0 (Dp)	18.2	4.5	—	—	—	—	22.7 (22)
Kunming – Xining temple	*Prunus* (*Cerasus*) sp.	May 2017	8.4 (Dp)	5.2	4.6	—	—	—	—	9.8 (504)
Fumin	*Myrica rubra*	June 2017	0.0	6.8	2.3	—			—	9.1 (88)
Fumin mountain	*Prunus* (*Cerasus*) sp.	June 2017	92.2 (Dp)	16.8	3.6	—	0.2	—	—	20.6 (552)
Fumin mountain	*Princepia utilis*	June 2017	92.5 (Dp)	9.5	—	—	—	—	—	9.5 (74)
**China - Beijing**										
Jiu Mountain	*Prunus* (*Cerasus*) sp.	June 2016	2.3 (Dsp)	0.2	1.0	—	0.04	—	—	1.2 (8114)
Lija Farm	*Prunus* (*Cerasus*) sp.	June 2016	0.0	1.6	1.6	—	—	—	—	3.3 (366)
Yiangtai Mountain	*Prunus* (*Cerasus*) sp.	June 2016	0.0	10.8	19.9	—	—	—	—	30.7 (251)
**China - Sichuan**										
Dazhou	*Prunus* (*Cerasus*) sp.	May 2016	−(Dp)^#^	2.7	15.2	—	0.4	2.1	—	20.4 (816)
**China - Hubei**										
Xiaoguan	*Coriaria nepalensis*	June 2016	100.0 (Dsp)	9.7	—	—	—	3.2	—	12.9 (31)
**China - Jilin**										
Changchun	*Vaccinium* spp.	Aug 2017	0.0	—	—	—	—	—	—	0.0 (2298)
Wanliang	*Rubus* sp.	Aug 2017	0.0	—	—	—	—	—	—	0.0 (15)
Quanyang	*Rubus* sp.	Aug 2017	0.0	—	—	—	—	—	—	0.0 (116)
Liaoyuan	*Vaccinium* sp.	Aug 2017	0.0	—	—	—	—	—	—	0.0 (147)
**Japan**										
Tokyo	*Prunus serrulata*	June 2015	0.0	7.5	0.2	—	1.2	—	—	8.9 (402)
Tokyo	*Prunus serrulata*	June 2016	0.0	26.3	1.5	—		—	—	27.8 (205)
Nara	*Morus* sp.	June 2016	0.0	75.6	—	—	—	—	—	75.6 (127)
Tsukuba	*Prunus serrulata*	June 2016	0.0	4.4	—	—	—	—	—	4.4 (45)
Yoshigadaira	*Vaccinium* spp.	Aug 2017	0.0	—	—	—	—	—	—	0.0 (95)
Hasuike	*Vaccinium* spp.	Aug 2017	24.0 (Dsp)	9.9	1.8	—		2.5	—	14.2 (566)
Yamanouchi	*Prunus* (*Padus*) sp.	Aug 2017	7.7 (Dsp)	—	—	—	—	—	—	0.0 (13)

Only samples that produced at least one larval parasitoid and more than 10 adults (*D. suzukii* + parasitoids) and for which quantitative data on parasitism are available are shown. Samples with no parasitism are shown for 2017 only. Parasitism rates were calculated, for each sample, by dividing the number of individuals of one or all parasitoid species by the total number of parasitoid and *Drosophila* spp. adults that emerged from the sample.

*Percentage of other *Drosophila* spp. in the sample, based on emerging adult flies.

Dp = *Drosophila pulchrella*; Dsp = *Drosophila subpulchrella*. Other *Drosophila* spp. accounted for less than 1% in all samples and were not included in the calculations.

^#^*D. pulchrella* present but not quantified.

^†^Includes at least three species; see text.

Larval parasitism rates were highly variable, from 0 to 75.6%. The highest rates of parasitism were observed in Yunnan Province (China) and Nara prefecture (Japan). In contrast, parasitism seems to be lower in northern and eastern China, as shown by collections in Beijing, Jilin, Inner Mongolia, Jiangsu and Zhejiang Provinces, which did not yield parasitoids (Tables 1 and S1 and unpublished data). A possible explanation could be that *D. suzukii* is likely non-native in these areas and parasitoids may be less well adapted to cold winter and hot summer conditions experienced in these regions. Strong variations in parasitism were observed between nearby sites but also from year to year at the same sites. For example, parasitism in Japan on the same *Prunus serrulata* Lindley (Rosaceae) trees in Tokyo climbed from 9% in 2015 to 28% in 2016 at the same period of the year. These strong variations in parasitism are typical for insects that have short development times and many annual generations. Indeed, abiotic factors may affect hosts and parasitoids differently. Another factor that may affect the estimation of parasitism rates is the fact that parasitoids attack young larvae whereas we probably collected *Drosophila* spp. as eggs as part of the sample, i.e. before the attack of the parasitoids, which may result in an underestimation of the parasitism rate. On the other hand, parasitoids emerge later than *D. suzukii* and it cannot be ruled out that some samples contained empty fly pupae and immature parasitoids in host pupae. This, however, is rather unlikely because most larvae leave the fruits to pupate^10^ and we specifically sampled fruits on the plant that looked undamaged, to avoid collecting other *Drosophila* species.

Other data on parasitism rates of *D. suzukii* in Asia are scarce. Data on parasitism in South Korea have been recently published^29^ and parasitism rates of 0–17% have been reported, i.e. in line with our results from Northern/Eastern China. Another survey focused on the genus *Asobara* (Braconidae) in South Korea and Yunnan^30^, and found two species, *A. japonica* and *A. leveri* (Nixon), associated with *D. suzukii*, but it did not provide parasitism rates. Parasitism of *D. suzukii* has been studied more extensively in Japan but the information provided in the literature is mostly qualitative. Quantitative data were published from *Prunus serrulata* in Tokyo in 2015 and 2016^27^. These results are very similar to our findings because fruits were collected at the same time. Other quantitative data from the same site in Japan have been published^24^.

In the majority of samples, *D. suzukii* was accompanied by two congeneric species that are also able to attack fresh fruits, *D. pulchrella* at high altitudes in Yunnan and Sichuan Provinces (China) and *D. subpulchrella* in Japan and at lower altitudes in China (Beijing and Hubei Provinces). This confirms the geographic range of the two species described^31^.

To our knowledge, this is the first time that *D. pulchrella* and *D. subpulchrella* have been sampled for parasitism. However, these two species occur nearly always together with *D. suzukii* and pupae of the three *Drosophila* species are morphologically indistinguishable, hence it was impossible to determine from which host the parasitoids emerged. Several samples, including very large ones, contained only *D. suzukii*, from which we deduced that the parasitoids emerged from this host. In contrast, only one small sample gave rise to *D. subpulchrella* only and no sample provided exclusively D*. pulchrella*. Thus, the association of the parasitoids with these two species could not be ascertained. The only solution would be to keep all pupae singly and to identify pupae from which parasitoids have emerged using molecular tools^32^.

### Larval parasitoid species

#### *Ganaspis* cf. *brasiliensis* (Hymenoptera, Figitidae)

A figitid wasp of the genus *Ganaspis* was the most frequently reared parasitoid of *D. suzukii* in China and Japan, being present in all samples from which parasitoids emerged. It was also the species that reached the highest levels of parasitism in both countries. The same parasitoid was also reared from the sample in Hubei province, from which *D. subpulchrella* emerged without *D. suzukii*, suggesting that *G*. cf. *brasiliensis* also parasitizes this host (Table 1).

All specimens were morphologically similar to specimens from *D. suzukii* collected in South Korea and Japan and were identified as *G. brasiliensis* by one of the co-authors (MB) who had examined the specimens of all studies^26,28^. *Ganaspis brasiliensis* was also the most abundant parasitoid collected in South Korea^29^ and Japan^27^. By using molecular tools and behavioural studies, it has been suggested that *G. brasiliensis* may be a complex of cryptic species and/or host races with unique distribution patterns and host ranges^26^. Sufficient evidence for taxonomic assignment have not been provided, and hence, no new names were proposed for these lineages. However, it has been shown that the host race of *G. brasiliensis*, attacking *D. suzukii* is the ‘suzukii-specialised’ type of *Ganaspis xanthopoda*^24^. It has been suggested that this host race only attacks and develops on *D. suzukii*^24,26^ and possibly other species inhabiting fresh fruits such as *D. pulchrella* and *D. subpulchrella*. Its specificity is largely confirmed by our studies in quarantine conditions in Switzerland^33,34^. The same species is also being considered for release in USA, where the biology of a Korean population has recently been studied in quarantine conditions^35^. Future research, combining data from multiple gene regions in specimens from across a wide geographic area and cross-mating experiments are required to further elucidate the taxonomic status of *Ganaspis brasiliensis s.l*. In the meantime, the specimens collected in this study from *Drosophila* spp. in fresh fruits are referred to as *Ganaspis* cf. *brasiliensis*.

#### *Leptopilina japonica* (Hymenoptera, Figitidae)

*Leptopilina japonica* has been found in all regions of China and at two sites in Japan, but rarely reached high parasitism rates. Among the 20 sites where this species was collected, we observed parasitism above 10% at only six sites, and with a maximum rate of 34.5%. However, it was at least as abundant as *Ganaspis* cf. *brasiliensis* in Beijing, and more abundant in the single sample from Sichuan (Table 1). This parasitoid was already known from *D. suzukii* in Japan and Taiwan^23,27^ and was also reared frequently from *D. suzukii* in South Korea^29^. To our knowledge this is the first record of *L. japonica* in the People’s Republic of China. Two sub-species^23^, *L. japonica japonica* Novković & Kimura and *L. japonica formosana* Novković & Kimura, have been reported from Japan and Taiwan, respectively. Both sub-species were also found in South Korea^29^. Morphological observations suggest that most species collected during this study belong to the subspecies *L. japonica japonica*. However, at least one sample collected in Hasuike (Japan) in 2017 provided specimens of *L. japonica formosana* and not all specimens reared during this study were identified to subspecies level. *Leptopilina japonica* was successfully reared on *D. suzukii* in the laboratory^34^ and is also known to successfully develop in *Drosophila biauria* Bock & Wheeler and *Drosophila rufa* Kikkawa & Peng in Japan under natural conditions and in *Drosophila simulans* Sturtevant in the laboratory^23^. Its biology has been recently studied in quarantine conditions in USA^35^.

#### *Leptopilina* sp. (Hymenoptera, Figitidae)

Another Figitidae was reared in high numbers from bayberry fruits collected at two sites in Yunnan (China). While one sample yielded a mixture of *D. suzukii* and *D. pulchrella*, only *D. suzukii* emerged from the other. This parasitoid has also been successfully reared on *D. suzukii* in the CABI laboratory in Beijing (J. Zhang, unpublished data). It is morphologically very different from *L. japonica*, and a description is presently being prepared (Buffington *et al*., in prep).

#### *Asobara* spp. (Hymenoptera, Braconidae, Alysiinae)

*Asobara* is the third important genus of larval parasitoids of Drosophilidae worldwide^15^. At least three species of *Asobara* spp. have been collected, but usually in very low numbers. *Asobara* sp. TK1 has been found in Tokyo. This undescribed species could be specific to *D. suzukii*, based on a study of the capacity of eight *Asobara* species associated with *Drosophila* spp. in Japan to parasitize *D. suzukii*^25^. So far, *Asobara* sp. TK1 has only been collected in Tokyo^25,27^, but it has been suggested that *Asobara* sp. TK1 could be the recently described species, *A. triangulata* van Achterberg & Guerrieri, based on the molecular analysis of one specimen from Yunnan, China^30^.

Other species found in this study include *Asobara pleuralis* (Ashmead) and *A. mesocauda* van Achterberg and Guerrieri, reared from pupae of *D. suzukii* and possibly *D. pulchrella* collected in Sichuan and Yunnan. *Asobara pleuralis* has also been reared from *D. suzukii* and/or *D. subpulchrella* in Beijing. These two species are known to be associated with *Drosophila* spp.^30^, but not yet with *D. suzukii*, *D. pulchrella or D. subpulchrella*. Other surveys for parasitoids of Drosophilidae in Yunnan (China) and South Korea found *Asobara japonica* and possibly *A. brevicauda* van Achterberg & Guerrieri and *A. leveri* (Nixon) associated with *D. suzukii*^29,30^.

#### *Tanycarpa chors* (Hymenoptera, Braconidae, Alysiinae)

*Tanycarpa* species are also known as parasitoids of *Drosophila* flies^15^. However, no species have ever been recorded from *D. suzukii, D. pulchrella* or *D. subpulchrella*^36^. In this study, *Tanycarpa chors* Belokobylskij was obtained from two sites in China and one site in Japan (Table 1).

#### *Areotetes striatiferus* (Hymenoptera, Braconidae, Opiinae)

Opiinae are very common larval parasitoids of Diptera, but more frequently attack fruit-infesting Tephritidae and mining Agromyzidae^37^. However, parasitism of Drosophilidae has been occasionally reported^38^. A few specimens of *Areotetes striatiferus* Li, van Achterberg and Tan emerged from pupae of *Drosophila suzukii* obtained from two sites in Yunnan (China). At one site *D. suzukii* was the only host species to emerge. *Areotetes striatiferus* was previously known only from Hunan province (China) and its biology was unknown^37^. It is also the first time that a species of *Areotetes* is recorded as a parasitoid of a *Drosophila* species.

## Conclusions - Prospects for biological control

For the first time, a large survey for larval parasitoids of *D. suzukii* was carried out in several Chinese Provinces. These surveys, and those made in Japan, revealed that most populations are parasitized and a complex of at least eight parasitoid species has been identified. The most abundant species collected during these surveys, i.e. *Ganaspis* cf*. brasiliensis* and *L. japonica*, are similar to those observed in previous surveys in Japan^24,27^ and South Korea^29^. Except *Asobara* sp. TK1 that was already known from Japan^25,27^, all other species found in this survey have been recorded for the first time from *D. suzukii* and should be further investigated. However, current studies presently on the biology of the parasitoids^33^, in accordance with previous research^24,27^, suggest that our *Ganaspis* cf*. brasiliensis*, or the s*uzukii*-specialised-race of *Ganaspis brasiliensis*, is the most specific parasitoid among those associated with *D. suzukii*. Since it is also the most abundant parasitoid of *D. suzukii* in Asia, it is clearly the first candidate for introduction into Europe, North America and other regions invaded by *D. suzukii*. The fact that it also probably attacks two other species also found in fresh fruits in Asia, *D. pulchrella* and *D. subpulchrella*, suggests that it may be specific to fresh fruits rather than purely host specific. This would not prevent its introduction in Europe and North America since native Drosophilidae in these regions are not able to attack fresh, undamaged fruits. These surveys also showed that, in some East Asian regions, *D. pulchrella* and *D. subpulchrella* are nearly as common in fresh fruits as *D. suzukii*, and their introduction to other continents should be avoided at all costs. It remains to be discovered whether *Ganaspis* cf. *brasiliensis* would be able to colonise all invaded regions or whether it would be limited by climatic constraints. The highest parasitism rates were observed in sub-tropical and warm-temperate climates in Yunnan (China) and Japan. However, the fact that *Ganaspis* cf. *brasiliensis* was also rather abundant at a ski resort in Nagano Prefecture (Central Japan: Hasuike, 1490 m average T° in January: ca. −13 °C) suggests that it may establish in temperate regions in Europe and North America. Since larval parasitism of *D. suzukii* in invaded regions has not been reported, introduction of a parasitoid may help lower populations and reduce the need for other management methods. Nevertheless, before its introduction, completion of host specificity tests and resolution of the taxonomic status of *Ganaspis* cf. *brasiliensis* are needed.

## Methods

### Collection sites and methods

**Figure 1:**
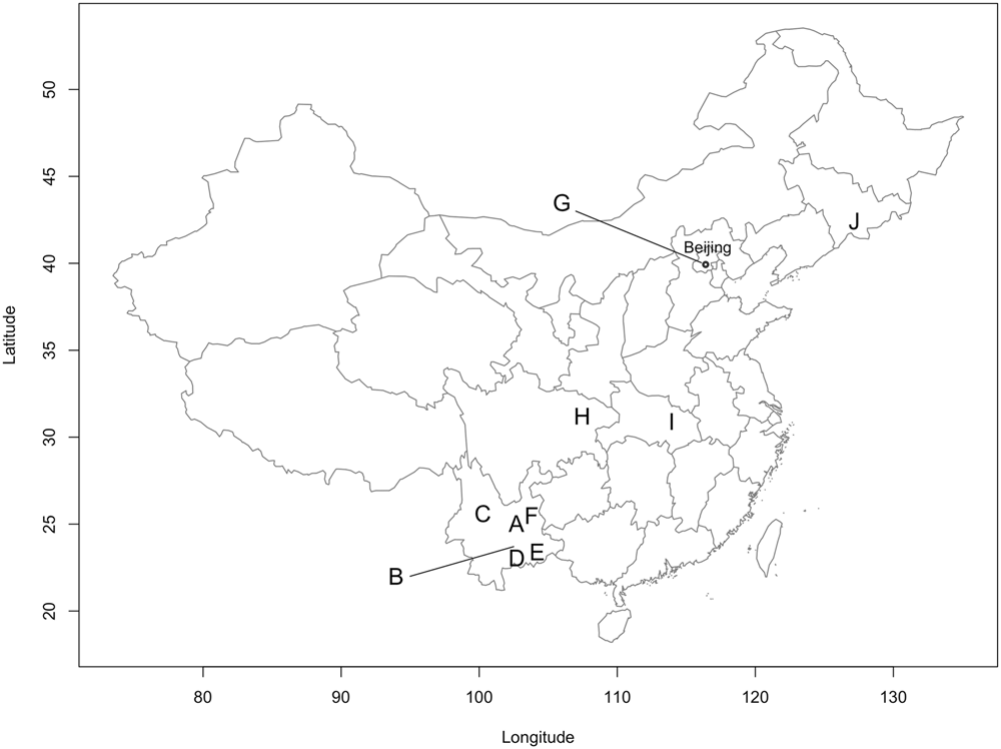
Geographic distribution of the sampling sites in China. A: Kunming - Fumin, B: Shiping, C: Dali, D: Panzihua, E: Wenshan, F: Qujing, G: Jiu Mountain - Lija Farm - Yiangtai Mountain, H: Dazhou, I: Xiaoguan and J: Jilin.

**Figure 2:**
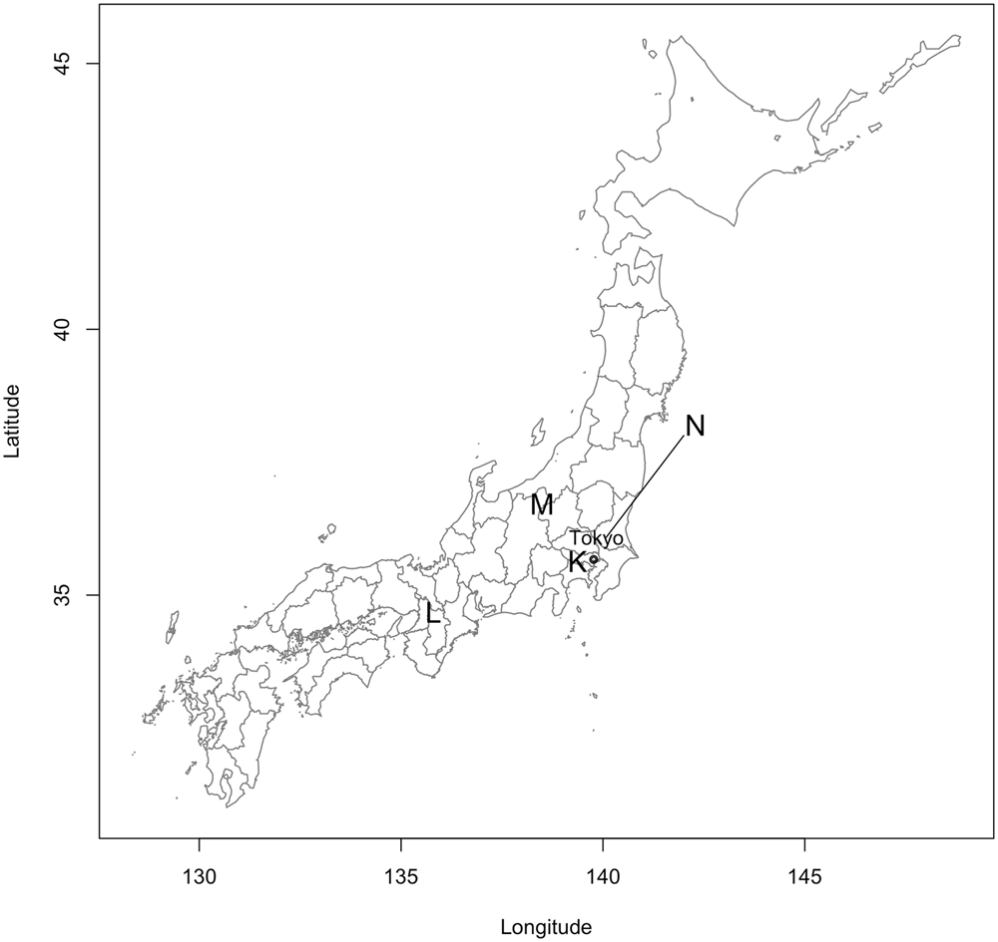
Geographic distribution of the sampling sites in Japan. K: Tokyo, L: Nara, M: Hasuike and N: Tsukuba.

Surveys for *D. suzukii* and associated parasitoids were carried out in China and Japan from 2015 to 2017. Fruits that could potentially host *D. suzukii* were collected at more than 100 sites in five prefectures in Japan and 12 provinces in China. Among these, seven sites in Japan (5 prefectures) and 29 sites in China (7 provinces) provided a sufficient number of *D. suzukii* and parasitoids (arbitrarily set at 10 individuals) to assess parasitism (Figs 1 and 2 and Table S1, Supplementary Information). Only fresh fruits that were still on the plant were sampled to avoid the collection of *Drosophila* spp. that prefer rotten or damaged fruits on the ground. The protocol to obtain *Drosophila* spp. and parasitoids varied slightly among years and regions but, in general, was as follows. Collected fruits were placed in a cooler box during their transport to the laboratory. Records of the location (GPS coordinates) and names of the collected fruit were annotated for each sample. In most cases, fruits were counted and then placed on a layer of slightly moist cellulose paper in plastic containers of various sizes, with ventilated lids. The boxes were inspected daily and *Drosophila* spp. and parasitoids that emerged were reared in a cage or placed in alcohol (96%). The cellulose paper was checked and moistened if necessary. After about a week, the paper was inspected and each fruit dissected to collect remaining drosophilid pupae as some larvae may have pupated inside the fruits. All pupae were placed in Petri-dishes on slightly moist cellulose paper. The Petri-dishes were then inspected daily. Emerged drosophilids and parasitoids were either put directly in alcohol or placed in cages for laboratory rearing.

### Identification of *Drosophila* spp. and parasitoids

*Drosophila suzukii*, *D. pulchrella* and *D. subpulchrella* were identified using keys^31,39^. Other *Drosophila* spp. were not determined to species level. Parasitoids were identified by MB, FRPF, MTK and MK using morphological characters and reference collections housed at the National Insect Collection (NIC), NMNH, Washington DC, and the Zoological Institute RAS of St Petersburg, Russia (ZISP). Vouchers for this study are housed at the NIC, the CABI collection in Delémont, the entomological collection of Natural History Museum Basel (NMBA) and the ZISP.

### Calculation of parasitism rates

Parasitism rates were calculated, for each sample, by dividing the number of individuals of one or all parasitoid species by the total number of parasitoid and *Drosophila* spp. adults emerged from the sample. Samples that contained more than 1% of *Drosophila* spp. unable to attack fresh, undamaged fruits, i.e. other than *D. suzukii*, *D. pulchrella* and *D*. *subpulchrella*, were discarded because it is supposed that these species have their own assemblage of parasitoids, specialised in rotten habitats, that may be different from those attacking *Drosophila* spp. specialised in fresh fruits”.

### Data Availability

The datasets generated during and/or analyzed during the current study are available from the corresponding author on reasonable request.

## Electronic supplementary material

S1
