# Supplementary material for: The parasitoid complex of D. suzukii and other fruit feeding Drosophila species in Asia
Source: Sci Rep. 2018 Aug 7;8:11839. doi: 10.1038/s41598-018-29555-8 (PMC6081417; doi:10.1038/s41598-018-29555-8)
Supplement: Supplementary file 1 — S1 [file 41598_2018_29555_MOESM1_ESM.docx]

# The parasitoid complex of *D. suzukii* and other fruit feeding Drosophila species in Asia

Pierre Girod^1,2^, Nicolas Borowiec^3^, Matthew Buffington^4^, Guohua Chen^5^, Yuan Fang^5^, Masahito T. Kimura^6^, Francisco Javier Peris-Felipo^7^, Nicolas Ris^3^, Hao Wu^5^, Chun Xiao^5^, Jinping Zhang^8^, Alexandre Aebi^9^, Tim Haye^1^ and Marc Kenis^1,*^

^1^ CABI, Delémont, Switzerland

^2^ Laboratory of Fundamental and Applied Research in Chemical Ecology (FARCE), Univ. Neuchâtel, Faculté des Sciences, Neuchâtel, Switzerland

^3^ INRA, Univ. Nice Côte d’Azur, CNRS, UMR 1355 “Institut Sophia Agrobiotech”, Sophia Antipolis, France

^4^ Systematic Entomology Laboratory, USDA Agricultural Research Service, Washington, D.C., USA

^5^ College of Plant Protection, Yunnan Agricultural University, Kunming

^6^ Hokkaido University Museum, Hokkaido University, Sapporo, Japan

^7^ Bleichestrasse 15, CH–4058 Basel, Switzerland.

^8^ MoA-CABI Joint Laboratory for Bio-Safety, Institute of Plant Protection Chinese Academy of Agricultural Sciences, Beijing, China

^9^ Laboratory of Soil Biodiversity, Univ. Neuchâtel, Faculté des Sciences, Neuchâtel, Switzerland

*** Corresponding author:**

E-mail address: m.kenis@cabi.org

**Supplementary information S1.** Details of the collections of *D. suzukii*, *D. pulchrella* and *D. subpulchrella* in China and Japan, 2015-2017. The table includes all collections in Table 1 plus collections from 2016 that did not provide parasitoids and for which the mortality of pupae was low (<20%).

| Locality | | | Fruit | Date of collection | Altitude (m) | Latitude N | | Longitude E | No. fruits | *D. suzukii* | *D. pulchrella* | *D. subpulchrella* | Parasitoids | In Table1 |
| --- | --- | --- | --- | --- | --- | --- | --- | --- | --- | --- | --- | --- | --- | --- |
| **China - Yunnan** | | |  |  |  | |  |  |  |  |  |  |  |  |
|  | | Kunming - YAU Campus | *Prunus cerasoides* | 03/06/15 | 1950 | | 25.1321 | 102.7484 | 200 | Yes | Yes | No | Yes | Yes |
|  | | Shiping | *Myrica rubra* | 06/06/15 | 1500 | | 23.7437 | 102.4816 | 250 | Yes | Yes | No | Yes | Yes |
|  | | Kunming - Snake mountain | *Corialia nepalensis* | 03/06/15 | 2000 | | 25.1322 | 102.7161 | 1400 | Yes | Yes | No | No | No |
|  | | Dali | *Prunus cerasoides* | 19/05/16 | 2020 | | 25.6103 | 100.2411 | 1450 | Yes | Yes | No | Yes | Yes |
|  | | Kunming – West Mountain | *Prunus (Cerasus)* sp. | 23/05/16 | 2200 | | 24.9683 | 102.6225 | 1360 | Yes | Yes | No | Yes | Yes |
|  | | Kunming - Snake mountain | *Corialia nepalensis* | 25/05/16 | 2000 | | 25.1183 | 102.7161 | 1200 | Yes | Yes | No | No | No |
|  | | Panzihua | *Prunus (Cerasus)* sp. | 31/03/16 | 1270 | | 23.0519 | 102.7394 | 572 | Yes | No | No | Yes | Yes |
|  | | Daheishan | *Prunus (Cerasus)* sp. | 01/04/16 | 1250 | | 23.0081 | 102.0650 | 310 | Yes | No | No | No | No |
|  | | Majiang | *Prunus (Cerasus)* sp. | 07/04/16 | 1550 | | 22.6986 | 101.2719 | 304 | Yes | No | No | No | No |
|  | | Fumin | *Myrica rubra* | 02/07/16 | 1980 | | 25.1475 | 102.5289 | 189 | Yes | Yes | No | Yes | Yes |
|  | | Honghe Prefecture,Shiping county | *Myrica rubra* | 02/06/16 | 1960 | | 23.7058 | 102.4650 | 350 | Yes | Yes | No | No | No |
|  | | Wenshan, Yanshan county | *Myrica rubra* | 25/06/16 | 1490 | | 23.5542 | 104.3475 | 295 | Yes | No | No | Yes | Yes |
|  | | Kunming – West Mountain, Dian Wei | *Myrica rubra* | 02/07/16 | 2010 | | 25.1475 | 102.5289 | 388 | Yes | No | No | Yes | Yes |
|  | | Dali | *Rubus sp.* | 03/07/16 | 2330 | | 25.5100 | 100.4414 | 1852 | Yes | Yes | No | No | No |
|  | | Dali | *Sambucus williamsii* | 23/07/16 | 2240 | | 25.5050 | 100.4356 | 1393 | Yes | Yes | No | Yes | Yes |
|  | | Kunming - Snake Mountain | *Solanum nigrum* | 28/07/16 | 2070 | | 25.1267 | 102.7147 | 713 | Yes | No | No | Yes | Yes |
|  | | Kunming,Xundian county | *Vaccinium spp.* | 29/07/16 | 1850 | | 25.5281 | 103.3294 | 1666 | Yes | Yes | No | No | No |
|  | | Yuxi, Mopanshan national Forest Park | *Fragaria nilgeerensis* | 18/08/16 | 2530 | | 23.9367 | 101.9883 | 168 | Yes | Yes | No | No | No |
|  | | Wenshan, Yanshan county | *Rubus sp.* | 22/08/16 | 1490 | | 23.5542 | 104.3475 | 26 | Yes | Yes | No | No | No |
|  | | Wenshan, Yanshan county | *Berchemia kulingensis* | 01/09/16 | 1490 | | 23.5542 | 104.3475 | 357 | Yes | Yes | No | No | No |
|  | | Wenshan, Yanshan county | *Rubus* sp. | 01/09/16 | 1490 | | 23.5542 | 104.3475 | 274 | Yes | Yes | No | Yes | Yes |
|  | | Qujing, Shizong county | *Lonicera maacki* | 04/09/16 | 2180 | | 24.6550 | 104.1706 | 198 | Yes | Yes | No | Yes | Yes |
|  | | Kunming - Botanical garden | *Lonicera maacki* | 23/09/16 | 1920 | | 25.1400 | 102.7408 | 209 | Yes | Yes | No | Yes | Yes |
|  | | Midu | *Rubus sp.* | 03/07/16 | 2370 | | 25.5089 | 100.4431 | 214 | Yes | Yes | No | No | No |
|  | | Midu | *Fragaria nilgeerensis* | 03/07/16 | 2371 | | 25.5089 | 100.4431 | 370 | No | Yes | No | No | No |
|  | | Kunming – West mountain | *Prunus (Cerasus)* sp. | 30/05/17 | 1930-2200 | | 24.7828 | 102.6167 | 2300 | Yes | Yes | No | No | Yes |
|  | | Kunming - Snake mountain | *Rubus* sp. | 31/05/17 | 2000 | | 25.1183 | 102.7161 | 410 | Yes | Yes | No | No | Yes |
|  | | Kunming - YAU | *Rubus ellipticus* (?) | 31/05/17 | 1950 | | 25.1444 | 102.7539 | 75 | Yes | Yes | No | Yes | Yes |
|  | | Kunming – Xining temple | *Prunus (Cerasus)* sp. | 31/05/17 | 1950 | | 25.1072 | 102.7167 | 1010 | Yes | Yes | No | Yes | Yes |
|  | | Fumin | *Myrica rubra* | 01/06/17 | Unknown | | From fruit market | From fruit market | 300 | Yes | No | No | Yes | Yes |
|  | | Fumin mountain | *Prunus (Cerasus)* sp. | 01/06/17 | 2550 | | 25.2053 | 102.4317 | 1848 | Yes | Yes | No | Yes | Yes |
|  | | Fumin mountain | *Princepia utilis* | 01/06/17 | 2550 | | 25.2053 | 102.4317 | 500 | Yes | Yes | No | Yes | Yes |
| **China - Beijing** | | |  |  |  | |  |  |  |  |  |  |  |  |
|  | | Xishanlinyu | *Prunus (Cerasus)* sp. | 21/06/16 | 50 | | 40.0350 | 116.2114 | 240 | Yes | No | No | No | No |
|  | | Jiu Mountain | *Prunus (Cerasus)* sp. | 22/06/16 | 200 | | 40.0350 | 116.0928 | 5456 | Yes | No | Yes | Yes | Yes |
|  | | Lija Farm | *Prunus (Cerasus)* sp. | 22/06/16 | 50 | | 40.0350 | 116.2114 | 1012 | Yes | No | No | Yes | Yes |
|  | | Yiangtai Mountain | *Prunus (Cerasus)* sp. | 22/06/16 | 350 | | 40.0686 | 116.0689 | 219 | Yes | No | No | Yes | Yes |
|  | | Yiangtai Mountain | *Morus* sp. | 22/06/16 | 350 | | 40.0686 | 116.0689 | 292 | Yes | No | No | No | No |
|  | | Miaofeng Mountain | *Morus* sp. | 22/06/16 | 430 | | 40.0431 | 116.0381 | 223 | Yes | No | No | No | No |
|  | | Miaofeng Mountain | *Prunus (Cerasus)* sp. | 22/06/16 | 430 | | 40.0431 | 116.0381 | 152 | Yes | No | No | No | No |
| **China - Sichuan** | | |  |  |  | |  |  |  |  |  |  |  |  |
|  | | Dazhou | *Prunus* (*Cerasus*) sp. | 05/05/16 | 420 | | 31.2739 | 107.4553 | - | Yes | Yes | No | Yes | Yes |
| **China - Hubei** | | |  |  |  | |  |  |  |  |  |  |  |  |
|  | Xiaoguan | | *Coriaria nepalensis* | 08/06/16 | 1000 | | 29.9392 | 109.3800 | 418 | No | No | Yes | Yes | Yes |
| **China - Jiangsu** | | |  |  |  | |  |  |  |  |  |  |  |  |
|  | Nanjing, Yinhewan garden | | *Myrica rubra* | 07/06/16 | 30 | | 32.0525 | 118.9250 | 120 | Yes | No | No | No | No |
| **China - Inner Mongolia** | | |  |  |  | |  |  |  |  |  |  |  |  |
|  | Tongliao airport | | *Prunus (Cerasus)* sp. | 14/07/16 | 180 | | 43.7464 | 125.4075 | 2411 | Yes | No | No | No | No |
| **China - Jilin** | | |  |  |  | |  |  |  |  |  |  |  |  |
|  | | Changchun - Jilin Univ. | *Vaccinium* spp. | 02/08/17 | 220 | | 43.7964 | 125.4075 | - | Yes | No | No | No | Yes |
|  | | Wanliang - Qingsheng | *Rubus* sp. | 03/08/17 | 630 | | 42.4253 | 127.1858 | - | Yes | No | No | No | Yes |
|  | | Quanyang | *Rubus* sp. | 04/08/17 | 770 | | 42.1694 | 127.4733 | - | Yes | No | No | No | Yes |
|  | | Liaoyuan | *Vaccinium* sp. | 04/08/17 | 270 | | 42.8833 | 125.1333 | - | Yes | No | No | No | Yes |
| **Japan - Tokyo** | | |  |  |  | |  |  |  |  |  |  |  |  |
|  | | Tokyo - Naganuma Park | *Prunus serrulata* | 03-07/06/2015 | 150 | | 35.6368 | 139.3647 | - | Yes | No | No | Yes | Yes |
|  | | Tokyo - Naganuma Park | *Prunus serrulata* | 01-02/06/2016 | 150 | | 35.6368 | 139.3647 | - | Yes | No | No | Yes | Yes |
|  | | Tokyo - Naganuma Park | *Morus* sp. | 31/05/2016 | 150 | | 35.6368 | 139.3647 | - | Yes | No | No | No | No |
| **Japan - Nara** | | |  |  |  | |  |  |  |  |  |  |  |  |
|  | | Nara | *Morus* sp. | 07-09/06/2016 | 170 | | 34.6688 | 135.8521 | - | Yes | No | No | Yes | Yes |
| **Japan - Ibaraki** | | |  |  |  | |  |  |  |  |  |  |  |  |
|  | | Tsukuba | *Prunus serrulata* | 09/06/2016 | 30 | | 36.0703 | 140.1209 | - | Yes | No | No | Yes | Yes |
| **Japan - Gunma** | | |  |  |  | |  |  |  |  |  |  |  |  |
|  | | Yoshigadaira - Shibu pass | *Vaccinium* spp. | 01/08/2017 | 1840 | | 36.6559 | 138.5483 | 350 | Yes | No | No | No | Yes |
| **Japan - Nagano** | | |  |  |  | |  |  |  |  |  |  |  |  |
|  | | Hasuike - Shiga Kogen | *Vaccinium* spp. | 01-02/08/2017 | 1490 | | 36.7189 | 138.4935 | 1560 | Yes | No | Yes | Yes | Yes |
|  | | Yamanouchi | *Prunus (Padus)* sp. | 02/08/2017 | 770 | | 36.7282 | 138.4424 | 500 | Yes | No | Yes | No | Yes |
